# Biopsy Characteristics, Subtypes, and Prognostic Features in 107 Cases of Feline Presumed Immune-Mediated Polyneuropathy

**DOI:** 10.3389/fvets.2022.928309

**Published:** 2022-06-24

**Authors:** Ninja Kolb, Kaspar Matiasek, Jana van Renen, Andrea Fischer, Yury Zablotski, Franziska Wieländer, Jasmin Nessler, Andrea Tipold, Rodolfo Cappello, Thomas Flegel, Shenja Loderstedt, Josephine Dietzel, Kirsten Gnirs, Kai Rentmeister, Stephan Rupp, Thilo von Klopmann, Frank Steffen, Konrad Jurina, Omar V. Del Vecchio, Martin Deutschland, Florian König, Gualtiero Gandini, Tom Harcourt-Brown, Marion Kornberg, Ezio Bianchi, Teresa Gagliardo, Marika Menchetti, Henning Schenk, Joana Tabanez, Marco Rosati

**Affiliations:** ^1^Section of Clinical and Comparative Neuropathology, Institute for Veterinary Pathology, Center for Clinical Veterinary Medicine, Ludwig-Maximilians-Universitaet Muenchen, Munich, Germany; ^2^Clinic for Small Animal Medicine, Center for Clinical Veterinary Medicine, Ludwig-Maximilians-Universitaet Muenchen, Munich, Germany; ^3^Clinic for Ruminants With Ambulatory and Herd Health Services, Center for Clinical Veterinary Medicine, Ludwig-Maximilians-Universitaet Muenchen, Munich, Germany; ^4^Department of Small Animal Medicine and Surgery, University of Veterinary Medicine Hanover, Hanover, Germany; ^5^North Downs Specialist Referrals, The Brewerstreet Dairy Business Park, Bletchingley, United Kingdom; ^6^Small Animal Department, University of Leipzig, Leipzig, Germany; ^7^Section of Neurology and Neurosurgery, Advetia Clinic for Small Animal Medicine, Paris, France; ^8^Specialty Practice for Veterinary Neurology and Neurosurgery, Dettelbach, Germany; ^9^Tierklinik Hofheim, Hofheim, Germany; ^10^Neurology Service, Department of Small Animals, Vetsuisse Faculty, University of Zurich, Zurich, Switzerland; ^11^AniCura Tierklinik Haar, Haar, Germany; ^12^Centro Veterinario Caleidos, Albisola Superiore, Italy; ^13^Neurological Referral Service, Berlin, Germany; ^14^Fachtierarzt für Kleintiere, Wiesbaden, Germany; ^15^Department of Veterinary Medical Sciences, University of Bologna, Bologna, Italy; ^16^Langford Veterinary Services, School of Veterinary Sciences, University of Bristol, Bristol, United Kingdom; ^17^AniCura Tierklinik Trier, Trier, Germany; ^18^Department of Veterinary Science, University of Parma, Parma, Italy; ^19^Palermovet, Veterinary Diagnostic Center, Palermo, Italy; ^20^Neurology and Neurosurgery Division, San Marco Veterinary Clinic, Veggiano, Italy; ^21^Tierklinik Lüneburg, Lüneburg, Germany; ^22^Neurology Section, Fitzpatrick Referrals, Godalming, United Kingdom

**Keywords:** feline, neuromuscular, histology, histopathology, tetraparesis, CIDP, GBS, outcome

## Abstract

Inflammatory polyradiculoneuropathy (IMPN) is one of the causes of sudden onset of neuromuscular signs such as para-/tetraparesis in young cats. Even though most cases have a favorable outcome, persistent deficits, relapses, and progressive courses are occasionally seen. As clinical presentation does not always appear to predict outcome and risk of recurrence, this study was initiated to screen for prognostic biopsy findings in a large cohort of histologically confirmed IMPN cases with clinical follow-up. In total, nerve and muscle specimens of 107 cats with biopsy diagnosis of presumed autoreactive inflammatory polyneuropathy and 22 control cases were reviewed by two blinded raters for a set of 36 histological parameters. To identify patterns and subtypes of IMPN, hierarchical k-means clustering of 33 histologic variables was performed. Then, the impact of histological parameters on IMPN outcome was evaluated *via* an univariate analysis to identify variables for the final multivariate model. The data on immediate outcome and follow-up were collected from submitting neurologists using a purpose-designed questionnaire. Hierarchical k-means clustering sorted the tissues into 4 main categories: cluster 1 (44/129) represents a purely inflammatory IMPN picture, whereas cluster 2 (47/129) was accompanied by demyelinating features and cluster 3 (16/129) by Wallerian degeneration. Cluster 4 (22/129) reflects normal tissues from non-neuropathic control cats. Returned questionnaires provided detailed information on outcome in 63 animals. They were categorized into recovered and non-recovered. Thereby, fiber-invasive infiltrates by mononuclear cells and mild fiber loss in intramuscular nerve branches correlated with higher probabilities of recovery. Remyelination in semithin sections, on the other hand, is correlated with a less favorable outcome. Animals grouping in cluster 1 had a tendency to a higher probability of recovery compared to other clusters. In conclusion, diagnosis of feline IMPN from nerve and muscle biopsies allowed for the identification of histologic features that were positively or negatively correlated with outcome.

## Introduction

Acquired polyneuropathies in cats are described as either of metabolic, toxic, paraneoplastic, immune-mediated, or idiopathic origin (1–14). Idiopathic, presumably immune-mediated, inflammatory polyneuropathy (IMPN) is sparsely reported in the current literature (1–3, 8, 15, 16), whereas in a previous study, we showed that 59% of all feline nerve biopsies submitted for diagnostic evaluation at a referral center were compatible with IMPN (6). Young animals of under 2 years of age are typically affected and present with sudden onset of neuromuscular signs, progressive course, and multiple episodes of relapses (1, 6, 15, 17, 18). Some studies suggest a breed predilection in Bengal cats (15), and a hereditary component has been proposed in Siberian cats (15, 16). Clinically, feline patients commonly present with progressive neuromuscular weakness, para- or tetraparesis, and show reduced to absent spinal reflexes (1–3, 6, 8, 16, 18). Involvement of cranial nerves is reported in a subset of patients (3, 6–8). Presumptive diagnosis of IMPN is based on the clinical presentation and electrodiagnostic evaluation, whereas histopathological evaluation of nerve and muscle specimens is required for confirmation and providing prognostic insights about the outcome (4, 18–20).

The limited literature describing the histopathologic changes in IMPN in cats reports most striking features of inflammation in nerve roots, especially in the ventral root (2), but dorsal root involvement and ganglioneuritis are also reported (7, 8) illustrating a heterogeneous motor and sensory neuropathy. Common histopathological findings in the peripheral nerves of IMPN-affected cats comprise mononuclear inflammatory infiltrates with or without signs of demyelination and remyelinating features (2, 6, 8, 15). Subsequently, muscle biopsies are affected by muscle atrophy of denervation type, and intramuscular nerve branches (IMNB) can display signs of inflammation or nerve fiber loss (2, 15). The outcome is variable ranging from full recovery, to partial recovery, and more rarely to fatal cases (1–3, 7, 8, 15, 18). The largest cohort study of Bengal cats by Bensfield et al. (15) reports a complete or partial recovery in 29 of 33 cats, and a fatal outcome in 4 cats during the first episode, followed by relapse in 12 feline patients with subsequent euthanasia in 2 further animals. Studies on large cohorts of feline patients with IMPN comparing histopathological changes and clinical outcome are currently lacking.

Immune-mediated polyneuropathies are presumed to be the veterinary correlate to the human Guillain–Barré syndrome (GBS) (3, 6, 7), the most frequent cause of acute paralytic neuropathy in people (21). Whereas the diagnosis in human medicine is entirely based on clinical, serologic, and electrodiagnostic evaluation (21), histopathological investigation of muscle and nerve biopsies is usually performed on companion animals (22). As IMPN in cats is a spontaneously occurring disease, much insight into the underlying immunobiology and prognostic indicators can be gained by the evaluation of nerve biopsies in this animal model.

The objective of this study was to perform a systematic histopathological investigation of nerve and muscle biopsies obtained from cats diagnosed with inflammatory neuropathy of presumably immune-mediated origin, and to correlate these findings with the clinical outcome. The following hypotheses were postulated: (1) IMPN can be subcategorized into histologic subtypes; (2) these subtypes correspond to diverse clinical courses and/or disease stage; (3) prognostic indicators correlating with the outcome can be identified from analysis of nerve and muscle biopsies.

## Materials and Methods

### Case Selection

Nerve and muscle biopsies of cats submitted for diagnostic purposes to the Section of Clinical and Comparative Neuropathology, Institute for Veterinary Pathology, Center for Clinical Veterinary Medicine, Ludwig-Maximilians-Universitaet Muenchen, Munich, Germany from 2011 to 2019 were retrospectively reviewed. Inclusion criteria for case eligibility comprised: (1) submission of at least one nerve and/or one muscle biopsy; (2) histologic diagnosis of inflammatory, presumed immune-mediated, neuropathy, based on the changes detected on peripheral nerve biopsies and/or terminal intramuscular nerve branches on muscle biopsies. Age-matched animals submitted to the necropsy service for reasons unrelated to this study and without a history of neurologic disease represented the control group. These latter underwent the same standard neuromuscular biopsy protocol for commonly biopsied sites including common peroneal or sciatic nerve, and cranial tibial muscle. Infectious agents were excluded by the referring neurologists according to the standard protocols for diagnostic workup of neuromuscular patients and to financial restrictions imposed by the owner.

### Tissue Processing

Surgical biopsies were sent overnight either as fresh and adequately cooled or formalin-fixed samples to the neuromuscular laboratory. After submission, epineurial and mesoneurial tissue was removed from the nerve samples. In case of unfixed tissue, nerves were fixed in 2.5% glutaraldehyde for 1 to 2 h depending on the thickness of the submitted material. Thereafter, nerves were rinsed and immersed in modified Sørensen phosphate buffer (pH 7.4) until further processing. One part of the sample was divided into four segments, contrasted to 1% buffered osmium tetroxide after Caufield for 1.5 to 2 h depending on thickness, and embedded in epoxy resin (Glycidether 100, Serva^®^, Heidelberg, Germany) after dehydration in an ascending alcohol series. Semithin sections (0.5 μm) were cut transversally as well as longitudinally and stained with toluidine blue and safranin O. The other part of the nerve sample underwent also osmium contrast enhancement with 2% buffered osmium tetroxide after Caufield for 1.5–2 h for nerve fiber teasing preparation. At least 30 fibers per nerve were prepared on each glass slide for detailed microscopic longitudinal analysis. In case, there was no formalin-fixed muscle specimen available, a part of fresh muscle was fixed in 10% neutral-buffered formalin and subjected to paraffin-embedding in longitudinal and transverse orientation, and slides were stained with hematoxylin–eosin (H&E), as well as Giemsa stain according to the standard protocols. Remaining fresh muscle samples were snap frozen in isopentane, cooled in liquid nitrogen (−130 to −150°C), and stored at −80°C until processing. Transverse cryosections (10 μm) were stained by the standard protocols including H&E, Engel's modified Gomori trichrome, periodic acid Schiff, oil-red O, cytochrome oxidase, and nicotinamide adenine dinucleotide dehydrogenase-tetrazolium reductase histochemistry, and fiber typing through immunolabeling of myosin heavy chains.

### Morphological Evaluation

Histological evaluation was performed by two blinded veterinarians experienced in neuropathology using light microscopy (Zeiss Axiophot^®^, Zeiss Instruments, Oberkochen, Germany). Cryosections and paraffin sections of skeletal muscle and nerve fiber teasing and semithin sections of peripheral nerves were investigated.

General algorithms for diagnostic evaluation of muscle and nerve biopsies were applied and subsequently implemented by a tailored grading scheme for nerve inflammation including 36 histological variables. Briefly, the scheme focused on topographic distribution and degree of severity of inflammatory infiltrates, axonal changes, Schwann cell pathology, nerve fiber degeneration, and regenerative features. Where applicable, semiquantitative scores were applied by grading each variable as summarized in Table 1. The dominant feature of each case was summarized and assigned to one of the following categories: infiltrative, degenerative, or mixed. Further details on the histomorphological criteria can be found in Table 1. Based on the nerve fiber teasing, the IMPN subtype after Gross et al. (6) was also classified (summarized in Table 2).

**Table 1:** Morphologic criteria applied for histological examination.

	**Material and histological processing**		
	**Nerve fiber teasing**	**Semithin sections of nerve**	**Muscle (FFPE and cryosections)**
Inflammatory features	Site of inflammatory infiltrates • Nodal • Paranodal • Nodo-paranodal • Schmidt-Lanterman cleft-directed • Diffuse • Diffuse predominantly nodo-paranodal Overall extensivity of fiber-directed inflammatory infiltrates • Score 0 (not present) • Score 1 (mild) • Score 2 (moderate) • Score 3 (severe) Degree of fiber-directed inflammatory cells • Score 0 (not present) • Score 1 (mild) • Score 2 (moderate) • Score 3 (severe)	Interstitial inflammatory infiltrates Fiber-directed inflammatory infiltrates Vasculitis	Inflammatory infiltrates in intramuscular nerve branches • Score 0 (not present) • Score 1 (mild) • Score 2 (severe)
Changes of the myelin sheath	Myelin sheath pathology Site of demyelination: • Nodal • Paranodal • Internodal • Inter-/paranodal • Segmental • Nodal-segmental • Paranodal-segmental • Mixed	Percentage of nerve fiber bundle affected by de-/remyelination: • ≤ 12,5% of nerve fiber bundle • up to 25% of nerve fiber bundle • 50–75% of nerve fiber bundle • ≥75% of nerve fiber bundle Degree of de-/remyelination • Score 0 (not present) • Score 1 (mild) • Score 2 (moderate) • Score 3 (marked) Onion bulb formation Schwann-cell pathology and/or hypertrophy	
Axonal features	Stage of Wallerian Degeneration: • 1 • 2 • 3 • 4 Distribution of Wallerian Degeneration • Continuous • Discontinuous Temporary stadium of Wallerian Degeneration: • Synchronous • Asynchronous	Degree of nerve fiber loss • Score 0 (not present) • Score 1 (mild) • Score 2 (moderate) • Score 3 (severe) Type of fibers affected by fiber loss: • Small fibers • Large fibers • Mixed Wallerian degeneration Post-resorptive macrophages Changes in axonal diameter • Score 0 (not present) • Score 1 (mild) • Score 2 (moderate) • Score 3 (severe)	Degree of nerve fiber loss in intramuscular nerve branches • Score 0 (not present) • Score 1 (mild) • Score 2 (moderate) • Score 3 (severe)
Regenerative features		Remyelination Regenerative clusters	Degree of muscle atrophy • Score 0 (not present) • Score 1 (mild) • Score 2 (mild to moderate) • Score 3 (moderate) • Score 4 (severe) Stadium of muscle atrophy • Score 0 (not present) • Score 1 (non-reactive muscle atrophy) • Score 2 (mild reactive changes) • Score 3 (fibrosis) Muscle fiber hypertrophy • Score 0 (not present) • Score 1 (mild) • Score 2 (moderate) • Score 3 (severe)

**Table 2:** IMPN subtypes according to Gross et al. (6).

**IMPN subtype**	**Histological features**
1	Cells are attached or enter the Schmidt-Lanterman clefts.
2A	Early invasive IMPN subtype 2 with marked demyelination, dysmorphic paranodes, paranodal retraction, and a only few cells located at the node of Ranvier.
2B	Infiltrative IMPN subtype 2 with cell-clusters in the area of the node of Ranvier.
3	Cells show an overall distribution along nerve fibers.
4	Mixed subtype with nodo-paranodal predominance.

### Clinical Questionnaire

An online questionnaire was sent to submitting veterinary neurologists to gather information about patient's history, onset of clinical signs, and course of the disease, neurological examination, results of the electrophysiological examination, imaging findings including MRI and CT, laboratory workup, and outcome. The survey (https://forms.office.com/Pages/ResponsePage.aspx?id=DQSIkW dsW0yxEjajBLZtrQAAAAAAAAAAAAN__iDTpglUQVVXTzN MNlc1UlpFUlo4UEdQOTNOVzRWMy4u) was performed using Microsoft Forms (Microsoft, Redmond, Washington, USA) in accordance with the General Data Protection Regulation, and with permission of the data protection officer of the Ludwig-Maximilians-Universitaet Muenchen, Munich, Germany. Participating clinicians were specialized in the field of veterinary neurology comprising board certified neurologists [European College of Veterinary Neurology and/or American College of Veterinary Internal Medicine (Neurology)] or an equal national qualification.

### Statistical Analysis

Hierarchical k-means clustering of 33 histologic variables was performed to identify similar histological patterns. Missing values were occasionally detected across the 33 variables and needed to be imputed, since cluster analysis requires complete dataset. Imputation was conducted *via* the missRanger approach, a non-parametric multivariate imputation by chained random forest algorithm with 1,000 trees (23). This method combines random forest imputation (24, 25) with predictive mean matching (26) and thus iterates multiple times until the average out-of-bag prediction error of the models stops to improve. After clustering, cluster features were analyzed to identify salient histological appearances. Individual animals were manually corrected, if the overall histological pattern was more suitable to a different cluster with confirmation of validity using Cohen's Kappa analysis. The impact of histological parameters on the outcome (recovery) and premedication was evaluated *via* univariate Bayesian generalized linear models to identify variables for the final multivariate model. Any variable having a *p* < 0.2 during the univariate test was selected as a candidate for the multivariate analysis (27). A stepwise variable selection among those variables leads to the final combination of predictors. All analyses were done by the R Statistical software (version 4.0.3).

## Results

### Signalment and Biopsy Site

Nerve and muscle biopsies of 107 affected cats were included in the study. Investigated cases originated from various countries in Europe including: Germany (*n* = 50), United Kingdom (*n* = 25), Italy (*n* = 12), France (*n* = 11), Switzerland (*n* = 8), and Estonia (*n* = 1). Age-matched control animals were solely submitted by veterinarians or private owners in Germany (*n* = 22).

The age of affected cats ranged from 3 months to 10.4 years with a median age of 0.9 years. About 30.8% of the cats (33/107 cats) were domestic shorthair cats (DSH), whereas 64.5% (69/107 cats) were purebred cats, 2.8% (3/107 cats) were mixed breeds, and for two cats' breed information was not available. Among purebred cats, British shorthair cats were overrepresented with 16.8% (18/107 cats) together with 12.1% of Bengal cats (13/107 cats), followed by 8.4% Maine Coon cats (9/107 cats), 4.7% of Persian cats (5/107 cats), and 3.7% of Siamese cats (4/107 cats). Other affected breed with 3 or less individuals comprised Siberian cat (*n* = 3), Birman cat (*n* = 3), Ragdoll (*n* = 2), Devon Rex (*n* = 2), Abyssinian cat (*n* = 2), and one of each Russian Blue, Turkish Angora, Savannah cat, Munchkin cat, Chartreux, Scottish Fold, Norwegian Forest cat, and a Thai cat. Gender distribution of the affected cats was 43 intact males (40.2%), 30 male neutered (28.0%), 26 intact females (24.3%), and 8 female spayed (7.5%).

Control samples were collected from 22 cats at 1.3 months to 11 years of age (median 4.3 years) consisting of 13/22 DSH, 6/22 purebred cats, and 3/22 mixed breed cats with 5 males, 7 male neutered, 7 females, and 3 female spayed cats.

Nerve biopsies were available in all of the cases and were collected from the hindlimb in 99 animals, from the forelimb in 3 animals, and from both in one cat. In 4 animals, the localization of nerve biopsy was not indicated. Muscle specimens were available in 105/107 cats and gained exclusively from the hindlimbs in 64 feline patients, from the forelimb in one animal, and from both in 34 animals. In 6 animals, information about localization of muscle biopsy was not provided.

### Histologic Features

#### Inflammatory Features Within the Nerves

The diagnosis of IMPN was based on the evidence of nerve fiber adhesive and/or invasive inflammatory infiltrates within the endoneurium, directed at the axons, nodes of Ranvier, and/or Schwann cells. Biopsies of cats without signs of inflammation in the intramuscular nerve branches and/or peripheral nerve biopsies were excluded from the study. IMPN was diagnosed in 105/107 animals based on findings in the main nerve trunk, and in 2/107 based on intramuscular nerve fiber branches. All animals showed inflammatory cells seen either in nerve fiber teasing (96/104; 92.3%), semithin preparations of the nerve (100/105 cats; 95.2%), or in the intramuscular nerve branches (69/79; 87.3%). Reduced numbers of animals result either from lacking of intramuscular nerve branches within muscle sections (26/105), and from lacking of either semithin sections or nerve fiber teasing preparations in case of limited nerve material submitted for diagnostic purposes. Only a subset of cases (38/105; 36.2%) showed fiber-invasive infiltrates in semithin sections, whereas interstitial inflammatory cells could be found in 87.3% (69/79, see above) of feline patients.

#### IMPN Subtypes According to Gross et al. (6)

The majority of cases with 42.1% (45/107 cats) presented with IMPN subtype 3 with diffuse inflammatory cell distribution. About 25.2% (27/107 cats) matched subtype 1 with focus of inflammation to the Schmidt-Lanterman clefts, and 16.8% (18/107 cats) were compatible with mixed subtype 4 with nodo-paranodal predominance. Only a small proportion with 5.6% (6/107 cats) fitted the criteria for the nodo-paranodal subtype 2, especially 1 animal with subtype 2a and 5 cats with subtype 2b. In 5 cats, inflammatory features presented only in semithin sections or in intramuscular nerve branches, and therefore, the subtype could not be determined. In 4 animals, IMPN type could not be defined as teasing preparations were not available due to low yield of nerve fibers from submitted material. In 2 patients, the subtype remained unclear.

#### Muscle Atrophy

Muscle samples were available for 105/107 cats. Of these, 86.7% (91/105) showed changes compatible with denervation atrophy. About 5.7% (6/105) of the cases had a mixed atrophy, and 7.6% (8/105) could not be further classified.

### Cluster Analysis

**Figure 1:**
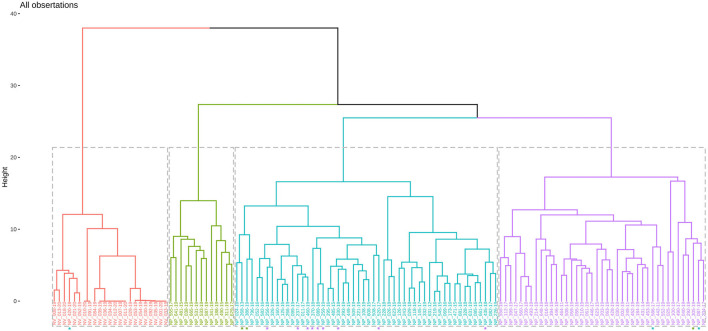
Dendrogram of hierarchical cluster analysis. Violet tree resembles cluster 1, turquoise tree cluster 2, green cluster 3, and orange cluster 4. Manual correction of single animals are shown with an asterisk (^*^) in the color of the final cluster.

Hierarchical k-means clustering sorted results of histologic scoring into 4 main clusters: cluster (1) (44/107) purely inflammatory IMPN; cluster (2) (47/129) IMPN accompanied by demyelinating features; cluster (3) (16/129) IMPN with signs of Wallerian degeneration; and cluster (4) (22/129) normal tissues from control animals. The cluster analysis dendrogram is shown in Figure 1.

#### Cluster 1: Purely Inflammatory IMPN

**Figure 2:**
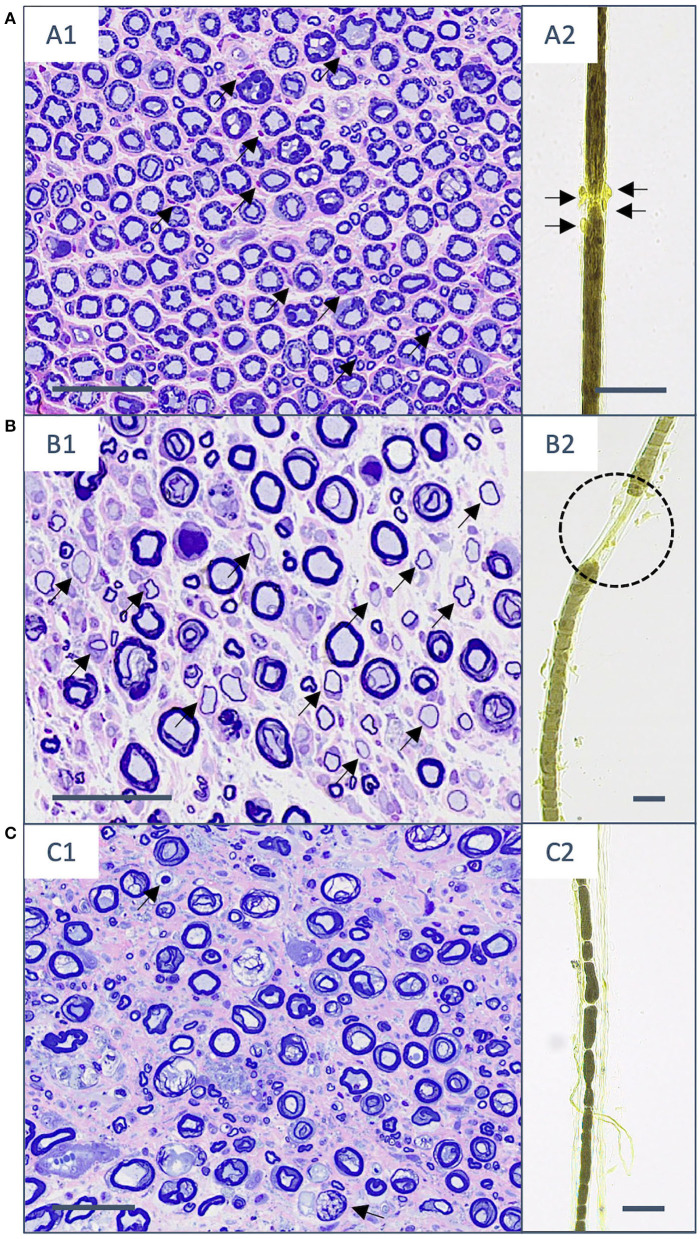
Histopathological changes in semithin sections (stained with toluidine blue and safranin O) and nerve fiber teasing preparations (contrasted with Osmium tetroxide) in the three different clusters. **(A)** Cluster 1. **(A1)** Transverse section of fibular nerve with interstitial and fiber-adhesive mononuclear infiltrates (arrows). **(A2)** The same cat shows mononuclear cell infiltrates directed to the nodo-paranodal area (arrows). **(B)** Cluster 2. **(B1)** The sciatic nerve with a moderately reduced number of myelinated fibers and multiple hypomyelinated fibers (arrows) on top of mononuclear interstitial and fiber-invasive infiltrates. **(B2)** Teasing preparations of the same animal shows paranodal retraction as a sign of demyelination (black dotted line). **(C)** Cluster 3. **(C1)** The peroneal nerve with markedly reduced number of myelinated fibers, endoneurial fibrosis, and signs of Wallerian degeneration (arrows). **(C2)** The fibular nerve of another cat with sings of stage II Wallerian degeneration. Scale bar: 50 μm.

Cluster 1 comprised 41.1% (44/107) of the cats. All cases showed lesions characteristic for IMPN as shown in Figure 2. Semithin sections of 93.2% (41/44) of the cats showed interstitial inflammatory cells, of these 24.4% (10/41) displayed additionally also fiber-directed infiltrates. Nerve fiber teasing preparations were available for 42/44 animals and showed inflammatory cell infiltration in 88.2% (37/42) of the cases. Semiquantitative measurement of the overall extensivity of inflammatory infiltrates in teasing preparations was mild in 45.2% (19/42), moderate in 16.7% (7/42), and severe in 26.2% (11/42) of the cases. Degree of inflammatory cell infiltrates per single fiber was graded mild in 54.8% (23/42), moderate in 21.4% (9/42), and severe in 11.9% (5/42) of the cats. A total of 3 of 4 cases of vasculitis observed in semithin sections were present in Cluster 1.

Affected breeds comprised 34.1% (15/44) DSH, 13.6% (6/44) Bengal cats, 11.4% (5/44) BSH, 9.1% (4/44) Maine coon cats, 6.8% (3/44) Persian cats, 4.5% (2/44) Siamese cats, one of each Siberian cat, Birman cat, Ragdoll, Devon Rex, Abyssinian cat, Scottish Fold, Thai cat, and also one mixed breed cat. In 2 cats, the breed was not known. There was no significant relationship [${\text{x}}_{\text{Pearson}}^{2}$(9) = 10.34, *p* = 0.32] between different breeds and specific clusters. Median age of the affected cats was 0.8 years (range 0.3–10.1 years). Gender distribution showed 54.5% (24/44) male or male neutered animals and 45.5% (20/44) female or female spayed cats.

#### Cluster 2: IMPN Accompanied by De-/remyelination

About 43.9% (47/107) of affected cats grouped in cluster 2. The hallmark of this cluster was the detection of de-/remyelination additionally to inflammatory infiltrates as shown in Figure 2. Demyelinating changes were seen in nerve fiber teasing preparations (33/46; 71.7%), and/or semithin sections of the nerve (32/45; 71.1%). The reduced number of animals in both preparation types results from lacking of teasing preparations in one cat or semithin preparations in 2 cats due to limited size of submitted nerve material. Demyelination could be localized in 33 cats *via* teasing preparation as paranodal (8/33; 24.2%), internodal (2/33; 6.1%), paranodal and internodal (1/33; 3.0%), segmental (17/33; 51.5%), nodal-segmental (1/33; 3.0%), paranodal-segmental (2/33; 6.1%), and mixed (2/33; 6.1%). The overall degree of de-/remyelination of individual nerve fibers could be semiquantitatively measured as mild in 62.5% (20/32) and moderate in 37.5% (12/32) of the cases with de-/remyelination present in semithin sections of the nerve. Remyelination was identified on semithin sections in 62.5% (20/32) of the cases. Extensiveness of de-/remyelinated fibers could be determined *via* semithin sections in 95.6% (29/32) of which 69.0% (20/29) with ≤12,5% of nerve fiber bundle affected, 31.0% (9/29) up to 25% of de-/remyelinated nerve fiber bundle, and 3.4% (1/29) of the cats with ≥75% of affected fibers. Onion bulb formation observed in semithin sections was present in 22.2% (10/45) of the cats. The remaining forth case of vasculitis was present in this cluster 2.

Breeds in this group comprised 27.7% (13/47) DSH, 21.3% (10/47) BSH, 12.8% (6/47) Bengal cats, 6.4% (3/47) Maine coon cats, 6.4% (3/47), Birman cat, 4.3% (2/47) Persian cats, 4.3% (2/47) mixed breed cats, one of each Siamese cat, Abyssinian cat, Russian blue, Turkish Angora, Savannah cat, Munchkin cat, Chartreux, and Norwegian Forest cat. Median age of cats in cluster 2 was 1 year (range 0.3–10.3 years). About 74.5% (35/47) of cats in cluster 2 were male or male neutered animals and 25.5% (12/47) female or female spayed.

#### Cluster 3: IMPN Accompanied by Wallerian Degeneration

About 15.0% (16/107) of the patients grouped in cluster 3. Wallerian degeneration was the unique feature in this cluster besides inflammatory infiltration shown in Figure 2. Wallerian degeneration was identified in nerve fiber teasing preparations in all but one animal (15/16; 93.8%) and in 37.5% (6/16) of the cases within semithin nerve sections. Stage of Wallerian degeneration could be determined based on nerve fiber teasing as follows: 1 in 6.7% (1/15), 2 in 13.3% (2/15), 3 in 26.7% (4/15), and 4 in 53.3% (8/15) of the affected cats. Along single teased nerve fibers, changes were considered as continuous in 80.0% (12/15) and discontinuous in 40.0% (6/15) of the cases. Temporal relationship of Wallerian degeneration among affected nerve fibers could be determined in 12 animals and defined as synchronous in (11.7%; 11/12) and asynchronous (8.3%; 1/12).

In cluster 3, 31.3% (5/16) were DSH, 18.8% (3/16) BSH, 12.5% (2/16) Maine coon cats, 12.5% (2/16) Siberian cats, and one of each Bengal cat, Siamese cat, Ragdoll, and Devon Rex. The median age of cluster 3 animals was 0.7 years (range 0.3–10.4). Male or male neutered cats in cluster 3 comprised 87.5% (14/16) and 12.5% (2/16) female cats.

### Online Questionnaire

A total of 73 survey answers were obtained, 3 were considered incomplete for further analysis, and 70 were valid surveys. Data on outcome were available in 63 animals, and information about therapies prior to biopsies collection was indicated in 70 survey responses. Outcome was classified into 2 categories, namely, recovered and not recovered. Recovery was defined as the state in which the cat could walk without assistance and could jump onto objects. Results of patient history, onset of clinical signs, course of the disease, neurological examination, results of the electrophysiological examination, imaging findings including MRI and CT, laboratory workup, and outcome are described by van Renen et al. (28).

#### Prognostic Histologic Parameters

**Figure 3:**
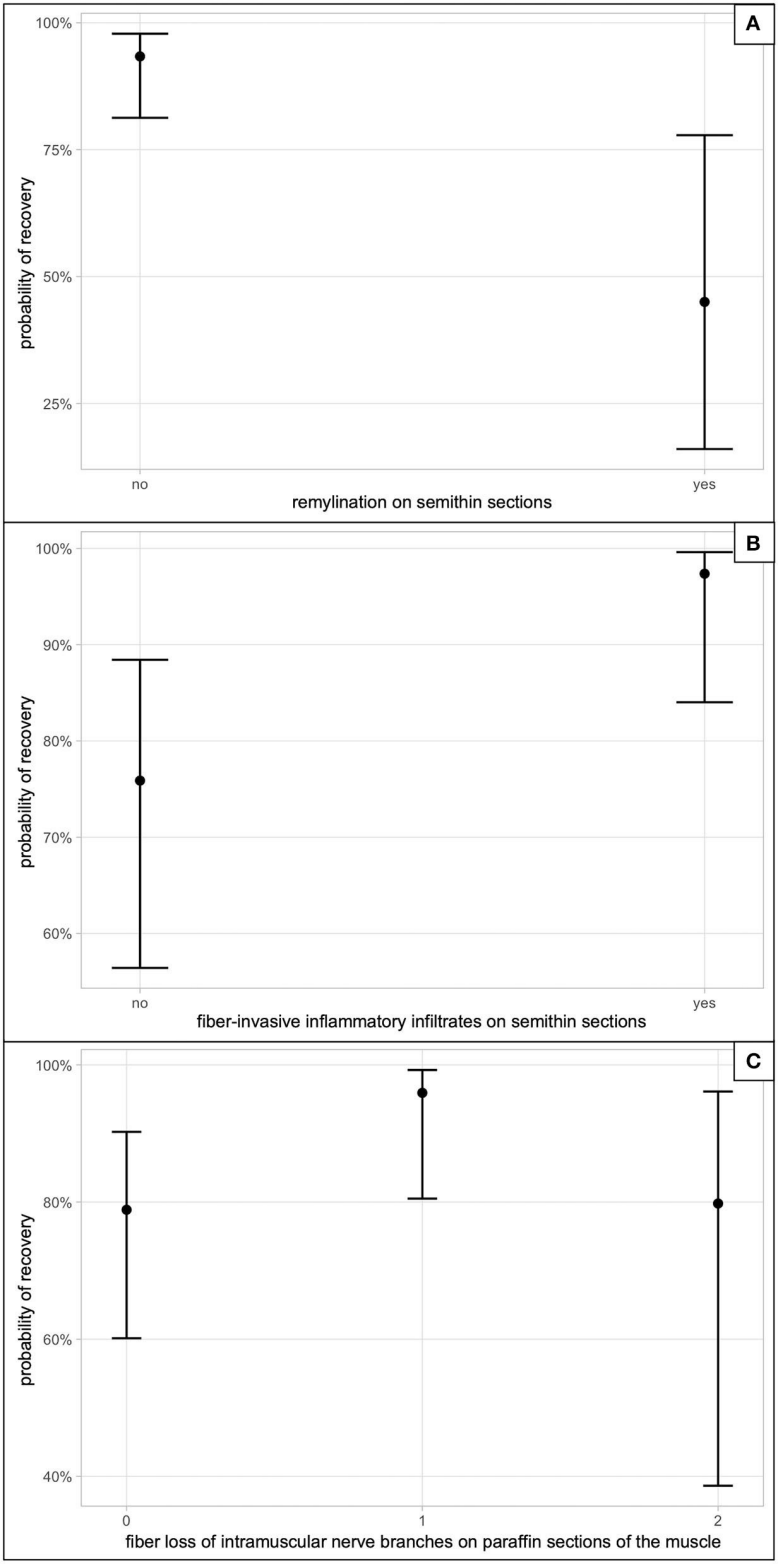
Probability of recovery in the multivariate model. **(A)** The impact on recovery, if remyelination on semithin sections was observed. **(B)** The correlation between presence of fiber-invasive inflammatory infiltrates on semithin sections and recovery. **(C)** Correlates the semiquantitative fiber loss score of intramuscular nerve branches seen on paraffin sections of the muscle in degree; 0, absent; 1, mild; 2, moderate; 3, severe (*n* = 0).

A univariate model with 31 of the histological parameters revealed 9 variables as significantly related to recovery of the animal (Table 3), which were then used for the final multivariate model. In the final model, remyelination in semithin sections was most significant related to recovery (*p* = 0.006), followed by fiber-invasive infiltrates in semithin sections (*p* = 0.022), and intramuscular nerve fiber loss (*p* = 0.045). The presence of remyelination was correlated with a lower probability of recovery compared to the absence of remyelinating features. When animals presented with fiber-invasive infiltrates, the probability of recovery was higher compared to cats where these infiltrates were mainly endoneurial. Fiber loss in intramuscular nerve branches observed in muscle sections was inhomogeneously correlated with probability of recovery. When fiber loss was moderate or absent, the probability of a favorable outcome was lower compared to mild fiber loss. Probabilities of recovery are shown in Figure 3.

**Table 3:** Significance of histological parameters in the univariate model.

**Histological phenomenon**	***P*-value**
**Paraffin sections of the muscle**	
Nerve fiber loss in intramuscular nerve branches	0.035
**Semithin sections of the nerve**	
Remyelination	0.044
Wallerian degeneration	0.076
Fiber-invasive infiltrates	0.105
Degree of de-/remyelination	0.130
Regenerative clusters	0.166
**Nerve fiber teasing preparations**	
Extensivity of inflammatory infiltrates	0.068
Degree of inflammatory infiltrates observed on single teased nerve fibers	0.103
Demyelination	0.198

### Histopathology Compared to Clinical Data

#### Therapies Prior to Biopsy Collection

About 62.9% of the cats (44/70) received medication prior to biopsy collection. Evaluation of the impact of medications on distribution and magnitude of inflammatory infiltrates on nerve biopsies did not reveal significant differences between pretreated and untreated patients. Medications included corticosteroids (all *p* > 0.395), non-steroidal anti-inflammatory drugs (NSAIDs) (all *p* > 0.601), and L-carnitine (all *p* > 0.123).

#### Outcome

**Figure 4:**
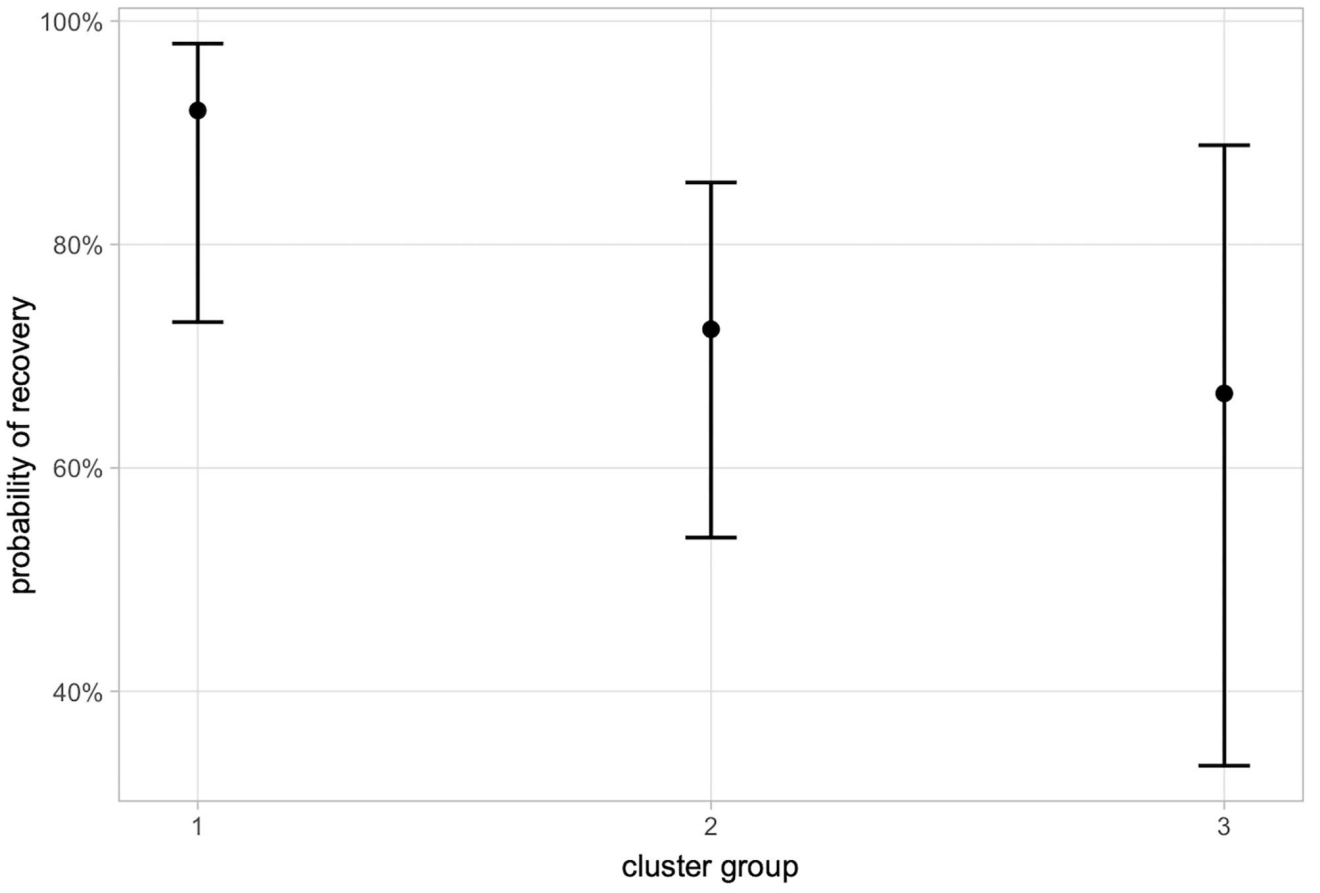
Probability of recovery in different cluster groups. 1, Purely inflammatory IMPN; 2, IMPN accompanied by de-/remyelination; 3, IMPN accompanied by Wallerian degeneration.

The probability to recover among the three clusters did not differ significantly, but showed a tendency toward a higher rate of recovery in cluster 1, compared to cluster 2 (*p* = 0.188), and 3 (*p* = 0.201) as shown in Figure 4. A correlation between the course of the disease (chronic: clinical signs lasting longer than 1 month; acute: clinical signs lasting less than 1 month) to the three different clusters could not be identified.

## Discussion

To date, this is the largest retrospective study on histopathological findings and clinical features of presumed IMPN in cats. Hierarchical cluster analysis defined three groups of IMPN with different histologic phenotypes: cluster 1 purely inflammatory, cluster 2 IMPN with additional demyelination, and cluster 3 where IMPN was accompanied by Wallerian degeneration. Salient histologic features with impact on patients' outcome were also identified through a multivariate analysis combining clinical and histologic data. Premedication with corticosteroids, NSAIDs, and L-carnitine had no significant impact on the magnitude of inflammatory features observed by neuropathologists on peripheral nerve biopsies.

In this cohort, 30.8% of cats were DSH and 64.5% purebreds with BSH being the most common breed accompanied by various purebreds not reported to be affected by IMPN. Previous studies described inflammatory/demyelinating/idiopathic polyneuropathy mainly in purebred cats (1, 3, 6, 15–17, 19), leading to the hypothesis of a hereditary neuropathy and/or a genetic predisposition in Siberian and Bengal cats (15, 16). Based on our findings, an inherited breed predisposition appears less likely as the number of breeds increased considerably with the present observations compared to previous descriptions, and biopsies were submitted from several referral centers distributed in 6 European countries. Breeds were distributed among all three different clusters with DSH as the most common. In a case series of Gerritsen et al. (3), DSH was also the most reported breed with 5/9 cases. Histopathology in DSH in other studies comprised also inflammatory infiltration, demyelination, and axonal degeneration with fragmentation in peripheral nerves (3, 8). Also, Bengal cats were found in all clusters, which fits to the previous study by Bensfield et al. where inflammation, demyelination, and axonal degeneration were described. In a Siberian cat, histologic evaluation of Crawford et al. (16) revealed a mononuclear cell infiltration within IMNB and the peripheral nerve. In our study, Siberian cats were present in clusters 1 and 3, but not in the de-/remyelinating cluster 2. In a case report of Jeandel et al., an Abyssinian cat showed loss of myelinated fibers and inflammatory infiltrates in the peripheral nerve. The two Abyssinian cats in our study were found in clusters 1 and 2, but not in cluster 3. Statistical analysis of a possible association of specific breed dispositions to the three different clusters revealed no correlation. Due to low numbers of animals in many breeds, further studies are needed to further exclude breed dispositions for specific histological subtypes.

As observed in previous investigations (1–3, 6, 8, 15, 17), IMPN is a disease mainly manifesting in young patients with 0.9 years as median age of onset in this study. However, 15/107 cats were older than 4 years in addition to what was described in the literature (6, 7, 18), we suggest to consider IMPN among differential diagnosis of neuromuscular signs even in adult cats. Gender distribution showed an overall predominance of male or male neutered animals (68.2%). This is in parallel with the biggest cohort study of Bensfield et al. (15) in Bengal cats, where 65% were male, and also Gross et al. (6) found 11/15 cats to be male or male neutered. Gender distribution in case series or case reports varies (1–3, 7, 8, 16, 17). Interestingly, gender distribution was close to equal in cluster 1, whereas in clusters 2 and 3, the gender gap was markedly skewed toward male sex. Similar findings are also recorded for human immune-mediated polyneuropathies such as Guillain–Barré syndrome (GBS) and chronic inflammatory demyelinating polyneuropathy (CIDP), where men are about 1.5 times more frequently affected compared to women (21). The reason for this gender predisposition remains unclear.

Diagnosis of IMPN was achieved from the evaluation of biopsies of the main peripheral nerve trunk and/or terminal nerve branches from muscle biopsies. Distribution of the lesions in inflammatory neuropathies can vary and show proximal (radiculitis), main nerve trunk (neuritis), and distal (terminal neuritis) changes (2, 6–8, 15–17). Multifocal lesions can severely affect one nerve fascicle and spare those neighboring as seen in a study of Aleman et al. (19) and further complicate diagnostic interpretation of peripheral nerve biopsies. Diagnosis of IMPN was obtained from main nerve trunk in 105/107, terminal nerve branches in 2/107, and from both sites in 67/79 animals. As in 87.3% of the investigated muscle biopsies intramuscular nerve branches were affected, muscle biopsy can help in diagnostic settings in case of multifocal distribution of the lesions. We conclude that a combination of nerve and muscle biopsies provides the highest yield of diagnostic samples in case of IMPN. Influence of medical treatments prior to biopsies collection and the yield of diagnostic features on submitted neuromuscular samples was also evaluated. Patients are often referred to veterinary neurologists with some delays from the onset of clinical signs, and empirical treatments are already established by the time of referral. In our population, corticosteroids, NSAIDs, and L-carnitine were administered prior to biopsy in 62.9% of the cases, but there was no significant impact on the diagnostic yield of inflammatory features, and a histologic diagnosis of IMPN could still be made. However, due to the retrospective nature of this study, more sound conclusions on the effects of prior therapies and their dosages (anti-inflammatory vs. immunosuppressive) could not be drawn.

Systematic evaluation of nerve biopsies identified changes comparable to those previously described including endoneurial mononuclear cell infiltration, demyelination, and axonal damage as indicated by Wallerian degeneration of nerve fibers (2, 3, 8, 15–17). Apart from unanimous identification of inflammatory infiltrates in peripheral nerves, in this cohort, as well as in past descriptions, there are some variabilities regarding distribution of inflammation along nerve fibers and damage of the Schwann cells and axons. According to Gross et al. (6), IMPN in cats and dogs can be subclassified based on nerve fiber teasing analysis of inflammatory cell distribution into 4 subtypes. In that study, the most common subtype in a cohort of 15 cats was subtype 4 mixed predominantly nodo-paranodal, followed by subtype 3 mixed cell distribution, subtype 1 Schmidt-Lanterman clefts distribution, whereas subtype 2 purely nodo-paranodal types were only present in one cat (6). Application of that scheme in this investigation revealed subtype 3 as the most common, followed by 1, 4, and 2 possibly reflecting a homogeneous density of antigenic triggering proteins along nerve fiber.

Hierarchical cluster analysis, applied in our study, sorted cases into three clusters based on histopathologic similarities. About 43.9% of affected cats grouped in cluster 2 making demyelination and Schwann cell pathology one of the main features of this condition besides inflammation. Demyelination is frequently mentioned also in previous reports (3, 6, 8, 17), and Bensfield et al. (15) report this feature in 11/17 Bengal cats, where peripheral nerves were available and pathologically affected. Wallerian degeneration characterizing cluster 3 was detected in 15% of our cases, and similarly, it has been rarely described in previous studies (3, 6, 7, 15). Cluster 1 with 41.1% of cats showed inflammatory changes in peripheral nerves only.

Histologic changes in our study are comparable to the human diseases GBS and CIDP, where inflammatory infiltrates in peripheral nerves and nerve roots, demyelination, and signs indicative of Wallerian degeneration can be found (29). Perivascular inflammatory infiltration can also be seen as additional features in few cases (29). Diagnosis of CIDP and GBS is based on clinical findings including muscle weakness, paresthesia, and reduced or absent tendon reflexes, electrophysiological examination, and CSF showing elevated protein content (30–32). In contrast to GBS, which has an acute onset, CIDP evolves over a course of more than 8 weeks (30).

At present, there is no clear reason and/or rational for patients grouping into one cluster instead of the other. We hypothesize that the pathomechanisms involved might be different, and that epitopes differ in topography along the axon-Schwann cell unit. As seen in human CIDP and GBS, multiple forms are described and characterized by clinical, serologic, and electrodiagnostic findings (30–33). Further studies are needed to elucidate and further characterize our observations.

Association of these clusters with signalment and course of the disease could not be identified. Though not statistically significant, recovery showed minor differences among clusters as cluster 1 had a tendency to a better outcome (Figure 4). Demyelination, nerve fiber loss, and Wallerian degeneration represent structural damage of the axon-Schwann cell unit requiring prolonged timespan to regenerate, and if regeneration is unsuccessful, residual deficits persist. In this vein, the absence of remyelination in semithin sections of the nerves was significantly correlated with a higher probability of recovery (*p* = 0.006). Remyelination [histologically seen as hypomyelinated nerve fibers (34)] follows segmental loss of Schwann cells and requires different signaling pathways, transcriptional regulators, and activation of epigenetic mechanisms (35, 36).

Mild fiber loss in intramuscular nerve branches was also associated with recovery (*p* = 0.045). Distally accentuated IMPN might have their target epitopes on the terminal portion of peripheral nerves close to the end organ. In this scenario, re-establishment of connectivity with the skeletal muscle benefit from (1) integrity of the perineurial sheaths, (2) less time required to fill a short gap, and (3) stimulation by trophic factors produced by the effector organs (37, 38). In line with these findings, fiber-invasive infiltrates in semithin sections were positively correlated with recovery (*p* = 0.022), and this might partly reflect the tendency to higher recovery seen in cluster 1.

In summary, our study shows three different clusters of IMPN based on histologic evaluation of nerve and muscle biopsies, characterized by inflammatory, demyelinating, and axonal changes. Age, gender distribution, and predominance of demyelinating features parallel findings of juvenile forms of CIDP.

Even when medication was already started before biopsies collection, diagnostic features of IMPN were still retained. Purely inflammatory changes are associated with a good prognosis, whereas chronic remodeling of the myelin sheath, as seen with remyelination, was negatively correlated with recovery.

## Data Availability Statement

The original contributions presented in the study are included in the article/supplementary material, further inquiries can be directed to the corresponding author/s.

## Ethics Statement

The animal study was reviewed and approved by Ethikkommission der Tierärztlichen Fakultät Ludwig-Maximilians-Universität München. Written informed consent was obtained from the owners for the participation of their animals in this study.

## Author Contributions

MR and NK designed, coordinated the study, and wrote the manuscript. NK, MR, and KM provided the data. AF, FW, MR, and KM designed the questionnaire. All authors read and approved the final manuscript.

## Conflict of Interest

The authors declare that the research was conducted in the absence of any commercial or financial relationships that could be construed as a potential conflict of interest.

## Publisher's Note

All claims expressed in this article are solely those of the authors and do not necessarily represent those of their affiliated organizations, or those of the publisher, the editors and the reviewers. Any product that may be evaluated in this article, or claim that may be made by its manufacturer, is not guaranteed or endorsed by the publisher.
